# FDG PET for therapy monitoring in Hodgkin and non-Hodgkin lymphomas

**DOI:** 10.1007/s00259-017-3690-8

**Published:** 2017-04-14

**Authors:** Sally F. Barrington, Regine Kluge

**Affiliations:** 1 PET Imaging Centre, King’s College London and Guy’s, King’s Health Partners, St. Thomas’ Hospital, Westminster Bridge Road, London, SE1 7EH UK; 2 Department of Nuclear Medicine, University Hospital of Leipzig, 0410 Leipzig, Germany

**Keywords:** Positron emission tomography, Lymphoma, Diagnosis, Therapy, Precision medicine

## Abstract

PET using ^18^F-FDG for treatment monitoring in patients with lymphoma is one of the most well-developed clinical applications. PET/CT is nowadays used during treatment to assess chemosensitivity, with response-adapted therapy given according to ‘interim’ PET in clinical practice to adults and children with Hodgkin lymphoma. PET is also used to assess remission from disease and to predict prognosis in the pretransplant setting. Mature data have been reported for the common subtypes of aggressive B-cell lymphomas, with more recent data also supporting the use of PET for response assessment in T-cell lymphomas. The Deauville five-point scale incorporating the Deauville criteria (DC) is recommended for response assessment in international guidelines. FDG uptake is graded in relation to the reference regions of normal mediastinum and liver. The DC have been validated in most lymphoma subtypes. The DC permit the threshold for adequate or inadequate response to be adapted according to the clinical context or research question. It is important for PET readers to understand how the DC have been applied in response-adapted trials for correct interpretation and discussion with the multidisciplinary team. Quantitative methods to perform PET in standardized ways have also been developed which may further improve response assessment including a quantitative extension to the DC (qPET). This may have advantages in providing a continuous scale to refine the threshold for adequate/inadequate response in specific clinical situations or treatment optimization in trials. qPET is also less observer-dependent and limits the problem of optical misinterpretation due to the influence of background activity.

## FDG PET for monitoring therapy and the development of the Deauville criteria

Monitoring of therapy in patients with lymphoma is one of the earliest published indications for PET using ^18^F-FDG [1–3]. Studies in the 2000s showed high FDG uptake in aggressive lymphomas, with rapid decreases in uptake occurring just two or three cycles into a planned course of chemotherapy lasting 4–6 months [4–7]. A rapid decline in uptake was found to be predictive of an excellent prognosis. Conversely, patients with persistent uptake on these early ‘interim’ scans had inferior prognosis, with progression-free survival (PFS) rates dependent on the subtype and stage of disease. PFS rates around 85–90% in larger series of patients with Hodgkin lymphoma (HL) have since been reported [8–13] and 70–90% in patients with aggressive non-Hodgkin lymphoma (NHL) [14–22] with a good response on interim PET. PFS in patients with a poor response on interim PET are inferior, at around 30–40% for HL treated with doxorubicin, bleomycin, vinblastine, dacarbazine (ABVD) chemotherapy [6, 13, 23], and 40–60% in patients with diffuse large B-cell lymphoma (DLBCL) [18, 20, 21]. Interim scans have become less discriminating in aggressive NHL in recent years, probably owing to improved treatment outcomes in patients treated with rituximab and better supportive care. This means that many patients with an adverse appearance on an interim scan still achieve remission at the end of treatment. However, PET/CT remains superior to CT assessment in this setting and is recommended for midtreatment imaging [24].

At the end of treatment, PET has proved to be highly predictive of disease remission, which is the goal of treatment. Negative predictive values of 90–100% have been reported for HL [11, 12, 25, 26] and aggressive NHL [14, 19, 27]. The positive predictive value is lower, because whilst FDG accumulates in residual tumour, it may also be taken up in inflammation induced by treatment. Published positive predictive values range from 50% to 100 [11, 12, 14, 19, 25–27] with variability again related to the initial disease prognosis. To minimize inflammatory uptake, imaging should be delayed for a minimum of 3 weeks, and preferably for 6–8 weeks, after chemotherapy at the end of treatment, 2 weeks after granulocyte colony-stimulating factor (GCSF) treatment and 3 months after radiotherapy (RT) [24].

PET was first included in response criteria alongside CT in 2007 as a way of confirming remission in patients with residual nodal masses [28, 29]. This led to the abolition of the response category ‘complete response unconfirmed’ (CRu) because PET is able to better differentiate patients with a ‘complete response’ (CR) from patients with a ‘partial response’ (PR). A residual mass of any size that was considered to be PET-negative was classified as CR, whilst a residual mass that was considered to be PET-positive was classified as PR or stable disease depending on the size on CT. This approach improved the accuracy of remission assessment in patients with high-grade NHL [30]. It was also accepted in the 2007 guidelines that PET-positive lesions could represent progressive disease, although the definition was still based on size on CT. These 2007 International Harmonization Project (IHP) criteria [28] restricted the use of PET to response assessment at the conclusion of therapy in patients with HL and DLBCL in clinical practice, with the use of interim imaging recommended only in clinical trials.

However, the potential for PET to guide therapy in lymphoma, including interim scans, was well recognized by haemato-oncologists [31]. The possibility to limit the amount of treatment given to patients likely to be cured, to reduce toxicity was appealing. The opportunity to escalate therapy in the minority of patients resistant to standard treatment was also an attractive option. Clinical trials began around 2005 in patients with HL followed by trials in patients with aggressive NHL to determine if PET-guided therapy could improve outcomes.

The need for standardization of imaging protocols and PET reporting in clinical practice and in trials was clearly recognized. Indeed, the application of PET for response-adapted treatment in lymphoma has been a key driver in the development of the imaging technique as a whole. Standardization of PET methods with the development of national research networks performing PET to commonly agreed quality assured standards [32, 33] has since been extended from lymphoma to other cancers. These methods are now generally recommended as best clinical practice [34–38].

## Application and use of the Deauville criteria

**Fig. 1:**
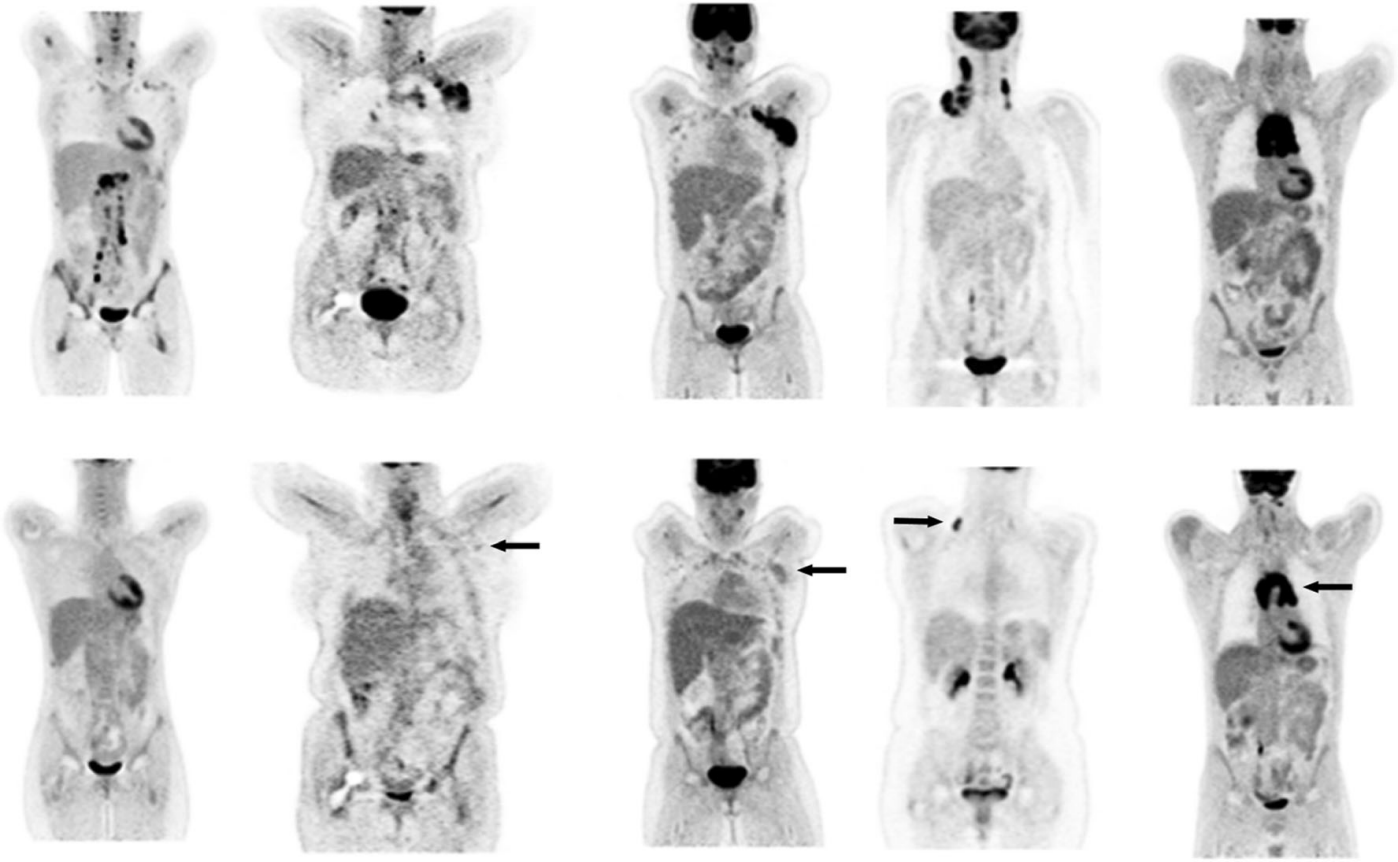
Coronal slices of PET images in patients at staging and at response with different grades of FDG uptake, from *left to right* corresponding to Deauville score 1–5

Initial reports mostly referred to PET-negative and PET-positive when reporting scans in lymphoma. Negative was defined for example as ‘no evidence of disease’ and positive as ‘any focal or diffuse area of increased activity in a location incompatible with normal anatomy and suspicious for residual disease’ on visual assessment [39]. The group at St. Thomas’ Hospital in London, however, acknowledged that varying levels of FDG uptake could be seen in reports of their initial experience, and in an attempt to grade residual uptake, introduced the concept of ‘minimal residual uptake’ (MRU) [5, 6, 40]. MRU was termed as ‘low-grade uptake of FDG (just above background) in a focus within an area of previously noted disease reported by the nuclear medicine physician as not likely to represent malignancy’ [40]. MRU is associated with varying prognosis depending on the stage, lymphoma subtype and timing of the scan during therapy [5, 6]. The group further refined this by grading uptake using a five-point scale initially referred to as the ‘South-East London Cancer Network score’ for patients with lymphoma. The score was used in the UK phase III trials sponsored by the National Cancer Research Institute (NCRI) testing response-adapted approaches in lymphoma and later by other research groups [41]. The first international workshop on PET in lymphoma was convened in Deauville to discuss solutions to deal with the lack of uniform and reliable criteria for interim PET scan interpretation [42]. It was concluded that this five-point scale should be applied for reporting scans using visual analysis and the additional value of standardized uptake value (SUV) analysis investigated. These criteria have since become widely known as the Deauville criteria (DC) and are incorporated into the Deauville five-point scale (Table 1, Fig. 1).

**Table 1:** The Deauville five point scale. The scale scores the most intense uptake in a site of initial disease, if present

Score	Definition
1	No uptake
2	Uptake ≤ mediastinum
3	Uptake > mediastinum but ≤ liver
4	Moderately increased uptake compared to the liver
5	Markedly increased uptake compared to the liver and/or new lesions
X	New areas of uptake unlikely to be related to lymphoma

Recognizing that it would be years before clinical trials testing PET response-adapted strategies would report, an international group from the Deauville workshop and subsequent workshops held in Menton, led by Professor Meignan and Dr. Gallamini, set out to validate the DC and to explore the ‘grey-zone’ area of MRU [42]. Cohorts of patient scans included in retrospective studies were collated and reported by readers from Europe and the US using the DC [23, 43]. These validation studies demonstrated good interobserver agreement and confirmed that the DC could predict outcome, confirming earlier results using less stringent criteria [4–7]. Training sets developed for use in clinical trials and prospective testing of agreement between national imaging core laboratories also showed good interobserver agreement in HL [44] and aggressive NHL [45, 46]. These studies also suggested refinements to the DC to further improve agreement.

In the 2007 IHP criteria, PET-positive scans were based on ‘purely visual assessment with focal or diffuse uptake above background in a location incompatible with normal anatomy’ although ‘mild and diffusely increased uptake at the site of masses ≥2 cm in diameter with intensity lower than or equal to mediastinal blood pool structures’ was regarded as PET-negative and likely due to post-therapy inflammation [29]. Semiquantitative analysis was regarded as unnecessary at that time.

The DC replaced IHP criteria in the updated international guidance in 2014 because of the need for a scale that reflected varying degrees of FDG uptake and the recognition that with modern PET/CT cameras uptake much higher than background could still predict a good prognosis [24]. The use of a graded scale also enables the threshold used to define a ‘negative’ or ‘positive’ scan to be adapted according to the clinical context or research question. In most situations where standard treatment will be given, a Deauville score (DS) of 3 represents a complete metabolic response (CMR). DS 3 has also proved to be the most reproducible threshold amongst reporters when interpreting PET scans in lymphoma patients. Furthermore, using the DC improves the positive predictive value of PET reporting compared to IHP and other criteria [15, 45, 47]. However, there is a caveat: if treatment is to be de-escalated, to avoid the risk of under-treatment some investigators have preferred to use the mediastinum (equivalent to DS 2) to define CMR or a ‘negative’ scan [9, 10, 25, 48]. It is important for reporters of PET to understand this and to report using the DS for objective interpretation and appropriate clinical management.

Refinements to the DC were included in the 2014 updated international recommendations for response assessment [24]. These guidelines stated that areas of the body with high physiological FDG uptake could have uptake higher than that of normal liver, yet still be considered to represent CMR. This applies to Waldeyer’s ring, bowel, spleen and bone marrow activated by chemotherapy and/or GCSF. In such circumstances, uptake at a site of initial disease that does not exceed that of surrounding normal tissue can be regarded as a CMR. It was also suggested that where a persistent focal abnormality was seen in the bone marrow in the context of a nodal response, evaluation with MR, biopsy or an interval scan should be considered as marrow changes may take longer to resolve [49]. Guidance was also given regarding (semi)quantitative assessment to assign DS 4 and 5 [24].

**Fig. 2:**
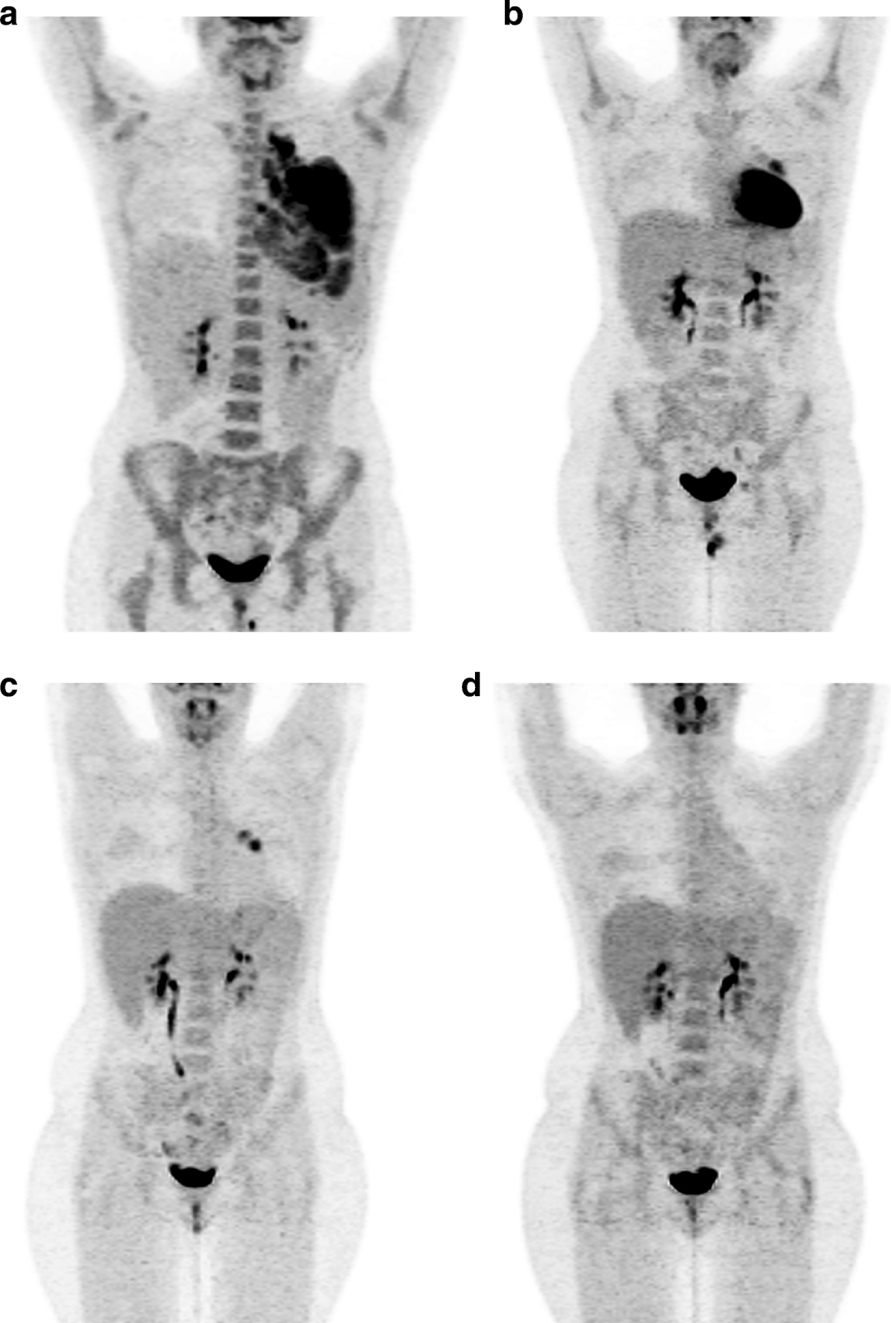
A 25-year-old woman with Hodgkin lymphoma. Maximum intensity projections of FDG PET. **a** The initial staging PET image reveals stage II disease. **b** The PET image after six cycles of chemotherapy (ABVD) shows PET-positive residual uptake at the left side of the mediastinum (Deauville score 5). **c** The PET image after involved field radiotherapy now demonstrates an additional focus of uptake (physiological heart uptake is suppressed on this scan). Biopsy revealed posttreatment changes only. **d** The interval PET image 12 months later shows resolution of uptake

**Fig. 3:**
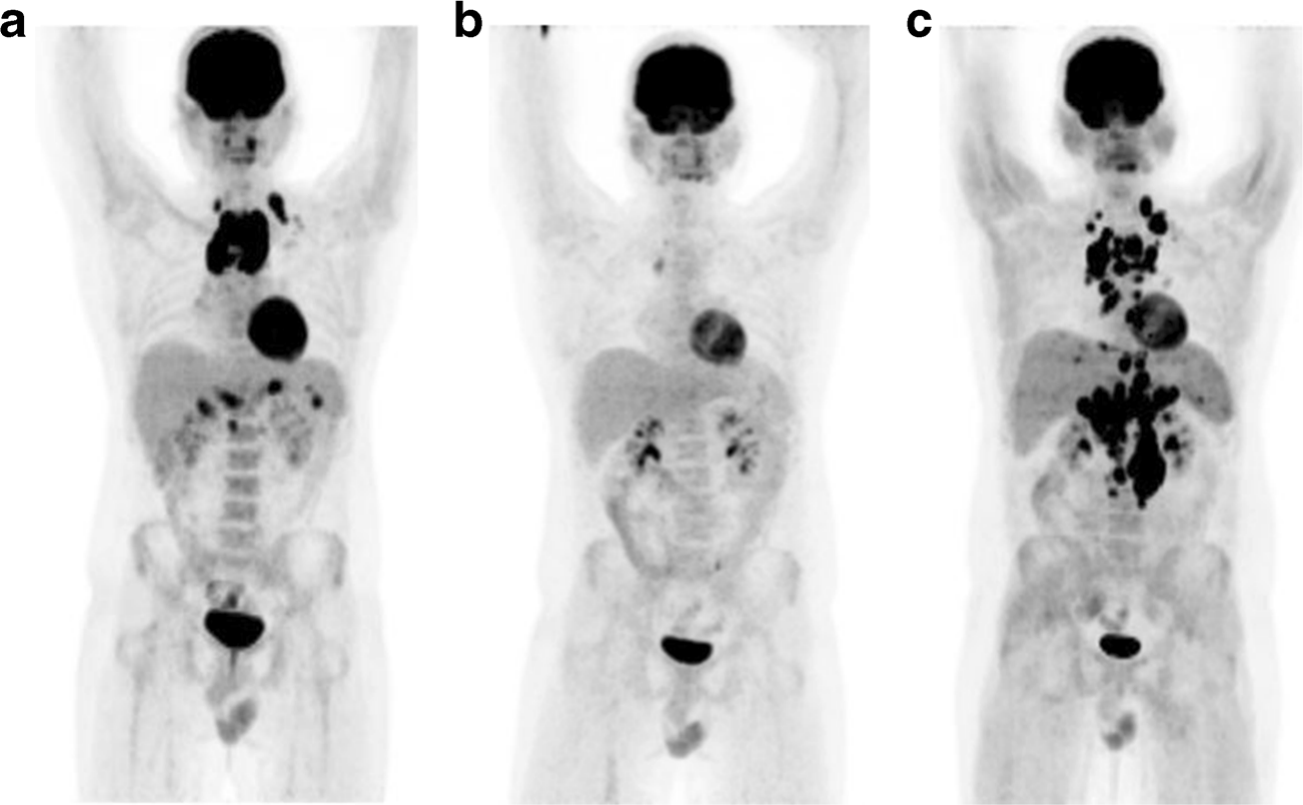
A 15-year-old boy with Hodgkin lymphoma. Maximum intensity projections of FDG PET. **a** The initial staging PET image reveals stage III disease. **b** The interim PET image after two cycles of chemotherapy (OEPA) shows PET-positive residual uptake at the right side of the mediastinum (Deauville score 4). **c** The PET image 4 months later shows extensive relapse of the disease (courtesy of Prof. Reinhardt, Pius Hospital Oldenburg)

Although initially developed for interim reporting, the DC were also recommended for response assessment at the end of treatment in the 2014 guidelines for staging and response assessment of all FDG-avid lymphomas [24, 49]. Data strongly indicate that PET/CT is more accurate than CT for remission assessment in follicular lymphomas (FL) treated with immunochemotherapy [50] as well as HL and aggressive NHL. However, because FDG uptake is not specific for lymphomatous residual disease (Figs. 2 and 3), biopsy is recommended if salvage treatment is considered, or at least an interval scan, if the clinical index of suspicion is low.

In the relapsed/refractory setting prior to autologous stem cell transplant, patients with a negative PET scan have better outcomes than patients with persistent uptake following high-dose chemotherapy in HL [51], DLBCL and FL [52]. Results of more recent studies have been reported according to DC with 3-year PFS in patients with DLBCL of 77% for DS 1–3 and 49% for DS 4 and 5 [53] and 3-year PFS in patients with FL of 75% for DS 1–3 and 43% for DS 4 and 5 [52].

The largest amount of published data relates to the use of PET in the common subtypes of HL, DLBCL and FL, which together account for 70% of lymphomas. Recent data also support the use of PET/CT for assessment of response in peripheral T-cell lymphomas [54–56] and extranodal NK/T-cell lymphomas [57, 58]. In NK/T-cell lymphomas, the DC combined with the presence/absence of Epstein-Barr virus (EBV) is an accurate predictor of response to chemotherapy and chemoradiotherapy [59]. Patients with a DS of 1 or 2 at the end of treatment and EBV-negative status have a 15% relapse rate after treatment compared to a 100% relapse rate in patients with a DS of ≥3 with EBV-positive status or a DS of 5 irrespective of EBV status. In cutaneous T-cell lymphomas, despite lower uptake in skin manifestations, PET has a role in detecting suspected visceral involvement [60] that may occur as the disease advances requiring systemic treatment, and in guiding biopsy in patients with suspected transformation to large cell disease [61].

The DC should now always be used to report response assessment in FDG-avid lymphomas and can be used to assign metabolic response categories (Table 2) [49]. Lymphoma subtypes are mostly FDG-avid, although some do not consistently take up FDG, including marginal zone lymphomas, small lymphocytic lymphoma and some cutaneous lymphomas [62]. CT is reserved for lymphomas with low or variable FDG avidity, or where PET is unavailable, and for evaluation of new agents in multiply relapsed disease, where disease control is more relevant than assessment of cure and data from PET are minimal.

**Table 2:** Using Deauville criteria to assign metabolic response categories

Category	PET/CT-based metabolic response
Complete metabolic response	Scores 1, 2 and 3^a^ in nodal or extranodal sites with or without a residual mass using the five-point scale.
Partial metabolic response	Score 4 or 5, with visually reduced uptake compared with baseline and residual mass(es) of any size. *At interim* these findings may suggest responding disease; *at end of treatment* these findings indicate residual metabolic disease. Bone marrow: residual marrow uptake > normal marrow but reduced compared with baseline (diffuse changes from chemotherapy allowed). If there are persistent focal changes in the marrow with a nodal response, consideration should be given to MRI, biopsy or interval scan.
No metabolic response	Score 4 or 5 with no significant change in uptake from baseline. *At interim or end of treatment*
Progressive metabolic disease	Score 4 or 5 with an increase in uptake from baseline and/or new FDG-avid foci consistent with lymphoma. *At interim or end of treatment*

Adapted from Cheson et al. [49]

^a^Score 3 probably represents CMR with standard treatment, but in trials where de-escalation is based on PET response, it may be preferable to consider score 3 as inadequate response to avoid undertreatment.

Emerging data suggest that patients with DS 5 have a significantly worse prognosis than patients with DS 4 at the interim assessment. In early stage HL, DS 5 is associated with a higher risk of progression or death from HL than DS 1–4, independent of pretreatment prognostic DS [63]. In advanced stage HL, DS 5 is also associated with inferior PFS [64, 65]. In primary mediastinal B-cell lymphoma, DS 4 is associated with a good prognosis, probably due to inflammation in the large residual mediastinal masses typical of this disease, but patients with DS 5 have a high chance of relapse [66].

## Quantification in response monitoring

Visual comparison of residual FDG uptake to uptake in the reference regions of the mediastinum and liver is the currently the defined standard for response interpretation in lymphoma and its clinical suitability has been proven, especially in HL. For clinical use, often a clear binary decision is desired—is the PET response adequate or inadequate? This interpretation, however, has to be made in the clinical context in relation to lymphoma type, stage and risk factors such as bulk or B-symptoms and the intensity of treatment given before and after the PET scan. Whilst in many patients with classical HL, hardly any residual uptake is present after two cycles of chemotherapy, patients with DLBCL nearly always have residual FDG uptake. Therefore, the degree of uptake considered adequate for a patient with DLBCL might be considered inadequate for a patient with HL. Minor diffuse residual uptake in a large bulky mass in a patient with stage IV HL also usually represents an adequate response after two cycles, as planned standard treatment with four to six more cycles will probably induce remission. The same degree of residual uptake might, however, be inadequate in a patient with stage II disease without additional risk factors, who is already at the end of standard treatment. Modification in the interpretation of the degree of FDG uptake may also be required for response-guided treatment. A lower level of FDG uptake might be preferred to define a so-called negative result in a clinical trial exploring de-escalation to avoid undertreatment. A higher level of uptake might be preferred to define a so-called positive result in a trial exploring escalation to avoid overtreatment [41].

Flexible criteria are, therefore, required. The change from the binary IHP criteria to the ordinal five-point DS has offered more flexibility to change the threshold between good and poor response according to the clinical context and/or treatment strategy. The intensity of residual uptake is, however, a categorical number whereby the full information can only be exploited using quantified methods. A continuous scale has the potential for optimal thresholds to be derived for normal/abnormal response for specific clinical situations or treatment optimization in clinical trials. Quantitative assessment is less user-dependent and circumvents optical misinterpretation due to the influence of background activity. It can be fully automated and permit easier comparison between centres, facilitating multicentre trials [67].

### Quantitative methods in oncology

Results from kinetic modelling or the Patlak-derived Ki correlate well with the SUV in oncological studies [68]. Römer et al. [69] showed good agreement between metabolic rate and SUV for chemotherapy response in NHL. Due to its complexity, full kinetic modelling is not typically undertaken for treatment response monitoring with FDG [70] and the SUV is the most widely used parameter for quantitative analysis. The SUV is the ratio between the radiotracer concentration in a voxel or group of voxels and the injected activity, divided by a normalization factor, which is in clinical practice is usually body weight. The SUV is readily available and well established in routine clinical work. It is, however, affected by a number of factors.

Differences in SUVs caused by differences in image acquisition parameters (scanner, scatter and attenuation correction, reconstruction algorithm) compromise the comparison of SUVs acquired at different centres. This is of special relevance in multicentre trials. Normalizing the tumour SUV to a reference region such as the liver in the same scan considerably reduces this problem, since in general, the effect is present in both regions and will at least partly, cancel each other out. Therefore, tumour-to-reference organ SUV ratios rather than direct SUV values should be used when comparing results from different scanners.

Partial volume effects can cause a marked underestimation of the true activity concentration within a tumour [71]. In phantom experiments, for a spherical lesion with a diameter 1.5 times the spatial resolution of the PET scanner at full-width at half-maximum, the measured maximum activity concentration is only about 60% of the true activity concentration [72]. In patients, during or after chemotherapy, the most metabolically active tumour residual is often very small, especially in responding lymphomas. Therefore, the residual uptake can be underestimated visually and quantitatively. The reconstruction algorithm may considerably influence this. Use of point spread function and time-of-flight reconstruction improves the detectability and increases the SUVs of small lesions [73] while the SUVs of reference organs such as the liver remain unaffected. In response evaluation, this effect may compromise both the visual Deauville scoring and the quantitative tumour-to-reference organ SUV ratios [74, 75], For response classification, it is therefore important to use standardized reconstruction methods as defined in the EANM procedure guideline for tumour imaging, especially in multicentre trials [36].

In most tumours, FDG uptake increases for at least 90 min after injection [71]. Standardization of the uptake time is very important, especially if the SUV of an individual patient will be monitored serially during the disease course. In contrast to tumour uptake, physiological FDG uptake in the liver or mediastinum does not increase over time. Thus, the tumour/reference SUV ratio will increase over time, requiring the use of a standardized time interval for determining tumour-to-organ ratios or the DS.

SUVs and ratios are strongly influenced by the size of the region. SUV_max_ is the uptake in the single voxel exhibiting the highest tracer uptake. SUV_max_ is easily available, has good interreader reproducibility and is relatively unaffected by partial volume effects. SUV_max_ is, however, highly dependent on the statistical quality of the images and is especially problematic with small voxel sizes and low uptake. SUV_mean_ is the mean uptake in a larger user-defined region. A fundamental biological question underlying the choice of region for SUV_mean_ is whether the total tumour volume or the most metabolically active portion is considered more important. Concepts of stem cell biology suggest that the most aggressive portion is more important, meaning that regions with the highest FDG uptake should be used for response assessment [70]. A variety of methods can be used to outline the volume of interest (VOI) giving different sizes and different SUV_mean_ values. SUV_peak_ is the average of the SUVs in a group of voxels surrounding the voxel with the highest activity. The idea is to combine the good interreader reproducibility and low influence of partial volume of the SUV_max_ with improved count rate stability. In 2009, Wahl et al. proposed PERCIST for better standardization of PET response criteria in solid tumours [70]. A SUV_peak_ in a (relatively large) spherical VOI of 1 ml is used, with semiautomatic positioning in the part of the tumour with highest activity. The mean SUV in a fixed-size VOI in the liver is used to measure reproducibility of serial data.

**Fig. 4:**
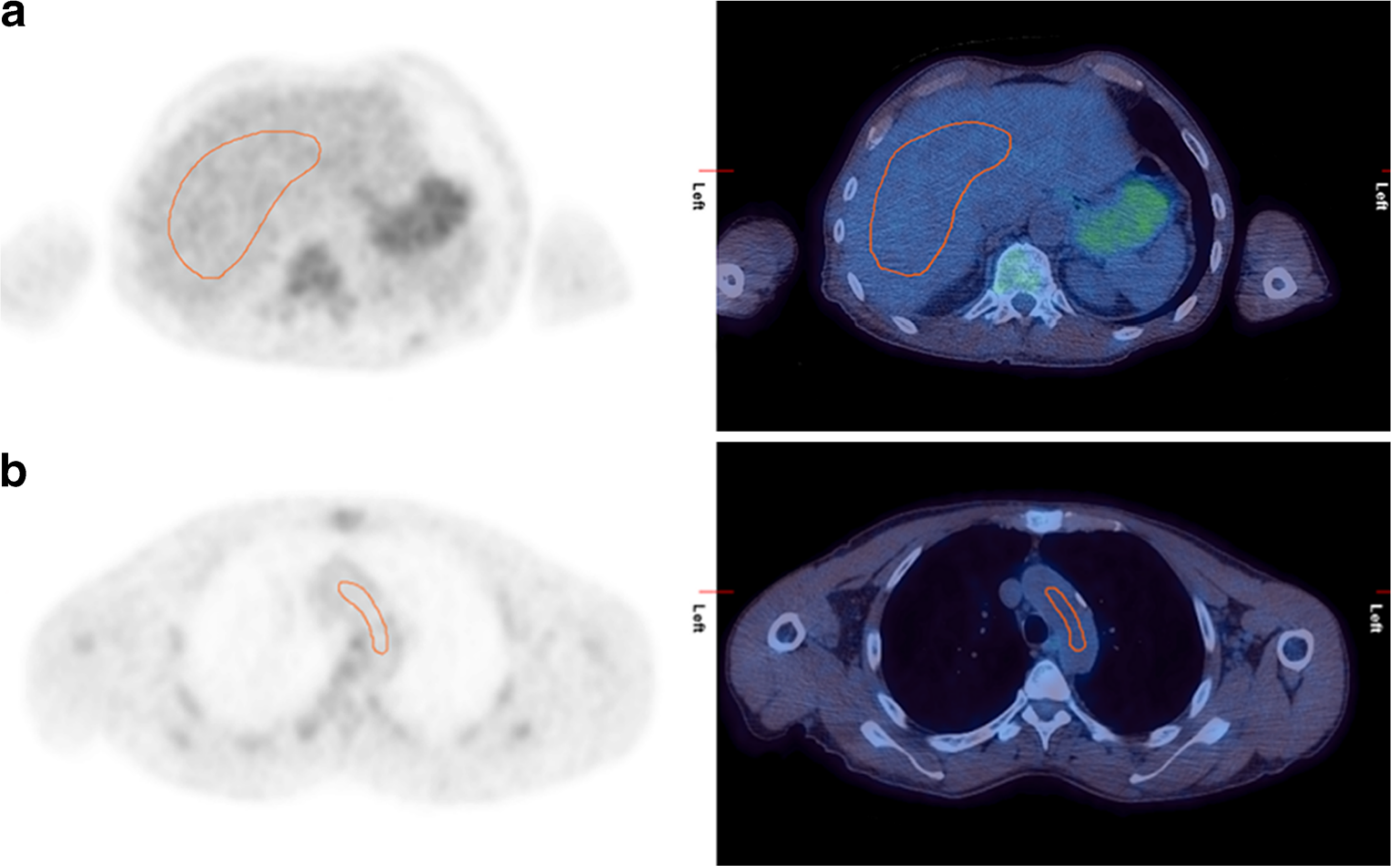
Axial slices demonstrating the St. Thomas’ method for assessment of maximum SUV within reference regions of the liver (**a**) and mediastinum (**b**) for confirmation of the Deauville score (courtesy of Dr. Victoria Warbey, PET Imaging Centre at St. Thomas’ Hospital London)

In the 2009 workshop on PET in lymphoma, it was proposed that SUV analysis should be explored [42] in addition to the visual DS. Visual comparison is influenced by the size and shape of the hottest residual uptake. It is not possible to look selectively at the voxel with maximal uptake or at a predefined peak area and to standardize the way different readers assess this. The level of residual uptake may be influenced by the adjacent background, giving misleading impressions of image contrast [24, 76]. Thus, in cases with residual uptake similar to the mediastinal or liver uptake, different observers may give different DS [43, 77]. The group at St. Thomas’ Hospital, London, recommends confirming visual evaluation by drawing regions of interest around residual area(s) of uptake, if present, and the normal mediastinum and liver. Regions of interest should be drawn for the liver, avoiding the edge and any individual/single ‘hot’ pixels likely to represent noise, sampling several axial slices to obtain a representative maximum liver SUV (Fig. 4) for comparison with the maximum SUV of the tumour. Regions in the mediastinum should be drawn in the arch of the aorta (or just above the aortic root in primary mediastinal B-cell lymphoma) [46] avoiding the vessel wall and any areas of calcification (Fig. 4). Comparison of maximum SUV in lesion and liver has also been proposed by other groups (‘rPET’ [78] and ‘Peking criteria’ [79]). Methods relying on SUV_max_ can be used with current commercial workstations, but are highly influenced by image noise.

Quantitative verification is of particular importance for discrimination between DS 4 and DS 5. Now with mature data published, DS 5 has been defined as three or more times the maximum SUV in the liver by the UK NCRI group in their trials and two or more times the maximum SUV in the liver by the French and Belgian Lymphoma Studies Association and Italian groups. Combining these, in the 2014 guidelines, it was recommended that DS 5 be applied to uptake two to three times the uptake in normal liver [24].

**Fig. 5:**
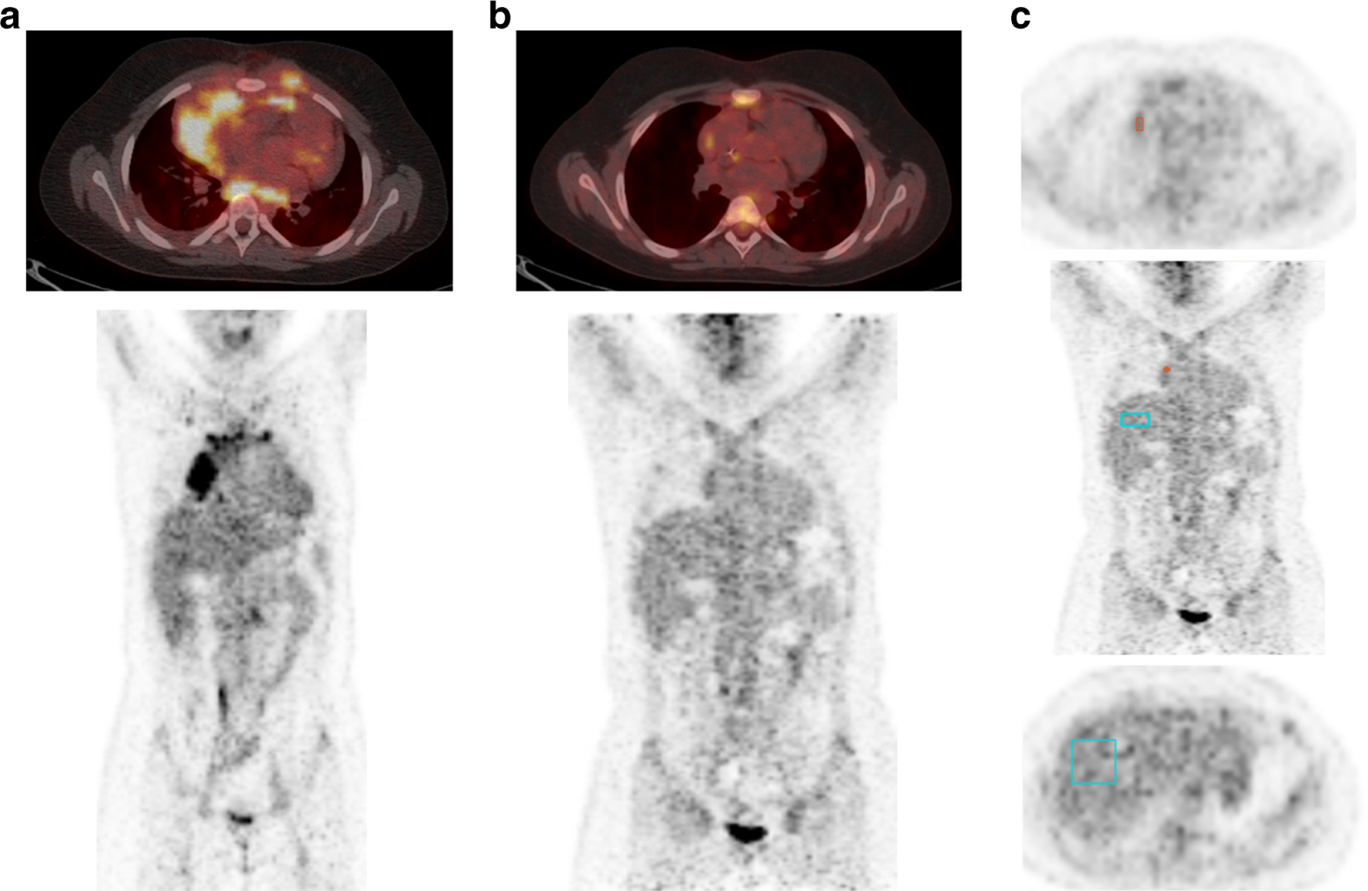
Initial (**a**) and interim FDG PET/CT (**b**, **c**) images in a patient with Hodgkin lymphoma. **b** Residual FDG uptake after two cycles of chemotherapy lies in the residual mass at the right side of the mediastinum. The intensity of the residual uptake is similar to the liver uptake; two independent readers scored this as Deauville scores 3 and 4, respectively. **c** Semiautomatic determination of the qPET value. *Cyan box* 30-ml VOI in the liver with SUV_mean_ 1.59; *small red box* four-voxel VOI in hottest part of the residuum with SUV_peak_ 2.34: qPET = 2.34/1.59 = 1.47, corresponding to Deauville score 4 (see Table 3)

In 2014, the group of Leipzig, Germany, proposed the qPET method as a quantitative extension of the DS [80]. In order to increase the count rate stability of the measured values, the SUV_peak_ of the residuum and the SUV_mean_ of the liver are used rather than SUV_max_. This approach corresponds to PERCIST recommendations for response assessment in solid tumours [70]. The qPET value is the quotient of the SUV_peak_ of the hottest residual over the SUV_mean_ of the liver. The most metabolically active part of HL residuals is typically very small. Therefore, a VOI of 1 ml [70] for the calculation of SUV_peak_ in solid tumours proved too large in HL. Hasenclever et al. [80] defined the SUV_peak_ as the average value in the maximum SUV voxel and the three hottest adjacent ones. The semiautomatic seed-growing algorithm (Hermes Medical Solutions, Stockholm, Sweden) is observer-independent. To characterize the average liver uptake, a cuboid VOI of 30 ml (edge length proportion 2:2:1) is positioned in the centre of the right lobe of the liver. This VOI size allows easy positioning even in small children but is large enough to give reproducible values for SUV_mean_, regardless of precise positioning within the liver (mean variation coefficient 3%). The authors independently determined DS and qPET values in 898 paediatric HL patients after two chemotherapy cycles and translated the ordinal (visual) Deauville categories to thresholds on the continuous qPET scale (Table 3). The method facilitates the use of the DC but enables clear decisions and reduces interobserver differences by standardizing regions and eliminating optical problems when comparing grey levels (Fig. 5). Additionally, the continuous scale offers a new approach to determining the best cutoff for normal/abnormal response in individual clinical trials. The histogram of qPET values in paediatric HL demonstrated a unimodal distribution with a pronounced peak suggesting ‘normal’ responses and a tail of outliers clearly indicating abnormal responses. Mathematical mixture modelling can be used to calculate the best cutoff. Based on this fitted model, patients with qPET values below 1.3 (corresponding to DS 1–3) among the 898 patients had a probability of 97% of belonging to the normal response group. The EuroNet clinical board decided to apply qPET in the EuroNet-PHL-C2 trial in paediatric HL which opened in 2015. The qPET method is semiautomatic, easy and fast, but currently is available only on Hermes workstations.

**Table 3:** Translation of semiautomatically derived qPET values to Deauville scores

qPET value	Deauville score
0	1
<0.95	2
0.95 to <1.3	3
1.3 to <2.0	4
≥2.0	5

The qPET value is the quotient of the mean standard uptake value of the four hottest connected voxels inside the tumour residual (SUV_peak_) and the mean SUV of the liver [80].

Another approach to the quantification of PET response is to determine ΔSUV_max_, i.e. the difference between the SUV_max_ of the lesions with the maximum uptake at baseline and after one or more cycles of treatment, representing the kinetics of tumour destruction [81]. This method has been explored mostly in DLBCL. Compared to a visual three-point score, Lin et al. showed an improved prognostic value of ΔSUV_max_ after two cycles of chemotherapy in DLBCL patients [82] with an optimal cutoff of −66%. Interestingly, similar results were obtained using an SUV_max_ on interim PET of 5.0, but this approach was not pursued further. After four cycles in the same patient group, a prognostic advantage of the ΔSUV_max_ method over visual analysis was no longer seen, even with the optimal cutoff of 70% [83]. Several trials have confirmed the suitability of the ΔSUV_max_ method with a cutoff of 66% after two cycles in DLBCL [43, 84]. After three or four cycles, this criterion is less successful [22, 85, 86]. In one trial involving DLBCL patients scanned after two or three cycles, the best ΔSUV_max_ cutoff was determined as 76% [85], and after three or four cycles, the best ΔSUV_max_ cutoff was as high as 92% [86]. In this later stage of treatment, the prognostic value of the ΔSUV_max_ method was not superior to the visual DS (regarding DS4/5 as PET-positive).

## Response-adapted treatment using FDG PET

In the last few years, clinical trials have begun reporting results using PET for response-adapted treatment. In HL, two trials have been published exploring whether RT can be omitted in patients with early-stage disease and a good response on interim PET. The trials enrolled 1,589 and 602 patients, respectively [9, 10]. The European H10 study had standard and experimental arms, with a PET scan performed after two cycles of ABVD (PET-2) in both arms. Patients in the standard arm received three to six cycles of ABVD, the duration depending on risk factors, followed by involved node RT (INRT). Patients in the experimental arm with uptake the same as or less than mediastinal uptake (equivalent to DS 2) on PET-2 continued with chemotherapy and were then randomized to receive either INRT or no further treatment [10]. In the UK RAPID study, with a similar design, patients were treated with ABVD with a PET scan performed after three cycles [9]. Patients with uptake the same as or less than mediastinal uptake (DS 2) stopped chemotherapy and were then randomized to receive involved field RT (IFRT) or no further treatment. Patients with uptake more than mediastinal uptake (DS3 or greater) received a fourth cycle of ABVD and IFRT.

The ethos of investigators in these two studies was slightly different. The H10 study was designed to test whether the omission of RT would be noninferior to standard treatment for early-stage HL, which is two to four cycles of ABVD chemotherapy and 20–30 Gy RT. The RAPID investigators, however, considered that omitting RT was likely to result in some worsening of disease control, which would be acceptable if balanced by improvement in overall survival (OS), by reducing the incidence of cardiopulmonary disease and second cancers, and by not irradiating all patients. The randomized arm in the H10 study for PET-negative patients was stopped early because of futility, i.e. the results indicated that 1-year PFS for patients who did not receive RT was inferior to that in patients receiving RT. The RAPID study continued to completion.

The results of the two studies are, however, similar. Both trials demonstrated inferior PFS in patients who did not receive RT. In the RAPID study, patients with a PET-negative scan who received RT had 3-year PFS of 97% and those who did not receive RT had a 3-year PFS of 91% (HR 2.39, *p* = 0.03 per protocol analysis). However, all patients with PET-negative scans had a good prognosis in both trials with 1-year PFS in patients with a PET-negative scan of ≥94% in the H10 study and a 3-year PFS of >90% in the RAPID study. The RAPID investigators concluded that patients with a negative PET scan after three cycles of ABVD could avoid RT but acknowledged that longer follow-up would be required to determine if this wold result in improved OS by reducing toxicity associated with RT. The decision as to whether an individual patient today should receive RT needs to be carefully considered in consultation with a radiation oncologist [87] and will probably depend on age, prognosis, fitness, disease distribution and the patient’s attitude to risk.

In advanced HL, the international UK-led RATHL trial explored de-escalating treatment in patients with a CMR on PET (defined as DS 1, 2 and 3) [8]. The study registered 1,214 patients. Patients were randomized after two cycles of ABVD to continue to six cycles of treatment with the standard four drugs or with bleomycin omitted, continuing with AVD for three to six cycles. Bleomycin is associated with respiratory side effects, especially in older patients. Patients did not receive RT in this trial, which is often given to sites of initial bulk as part of standard treatment. Three-year PFS was not significantly different between patients receiving ABVD and those receiving AVD (HR 1.13, *p* = 0.35), but patients receiving AVD had less infection, neutropenic fever and respiratory adverse events. The investigators concluded that patients with an early CMR can safely have bleomycin omitted from treatment in cycles three to six with reduced toxicity.

Patients with advanced HL are commonly treated with six cycles of ABVD in some parts of Europe and the USA. However, in other parts of Europe, six to eight cycles of bleomycin, etoposide, doxorubicin, cyclophosphamide, vincristine, procarbazine, prednisolone (BEACOPP) is preferred for first-line treatment. ABVD cures around 70–80% of patients, whereas BEACOPP cures 5–10% more patients but with a worse side effect profile including more acute haematological toxicity and increased risk of infertility and second malignancies [88]. Chemotherapy is usually followed by RT to sites of initial bulk.

The H10 and the RATHL studies attempted to evaluate whether BEACOPP chemotherapy could be reserved for patients with an early PET-positive scan, thereby reducing side effects overall.

In the H10 trial patients with early-stage disease and a PET-positive scan (DS 3, 4 and 5) were randomized to receive ABVD or BEACOPP then INRT [10]. In the RATHL trial all patients with advanced disease and a PET-positive scan (DS 4 and 5) were treated with BEACOPP chemotherapy but no RT [8]. The H10 study demonstrated significantly better PFS in PET-positive patients receiving BEACOPP and INRT than in patients receiving ABVD and INRT, with 5-year PFS of 91% and 77%, respectively. RATHL demonstrated 3-year PFS of 67% for PET-positive patients treated with BEACOPP compared with historical reports of PFS at 2–3 years of 20–30% in PET-positive patients with advanced disease treated with ABVD [7, 23]. Both trials suggested that patients with a PET-positive scan have better outcomes if escalated from ABVD to BEACOPP, sparing >80% of patients the side effects associated with BEACOPP.

The Italian HD0801 also used a PET response-adapted approach in patients with advanced disease after two cycles of ABVD [89]. Patients with PET-negative scans (*n* = 409) continued with ABVD, whereas patients with a PET-positive scan (*n* = 101) were treated with high-dose chemotherapy and bone marrow transplantation. Patients with a PET-positive scan had similar PFS to patients with a PET-negative scan with 2-year PFS of 74% and 81%, respectively, again suggesting that outcome can be improved with treatment escalation if early PET demonstrates chemoresistance to ABVD.

The German HD15 study used PET to decide whether patients with a residual mass ≥2.5 cm at the end of BEACOPP treatment should receive RT [25]. The more conservative mediastinal threshold (DS 2) was used to define response. PET was performed in 881 patients, 74% of whom had a negative PET scan and did not receive RT. Patients with a complete radiological response had the same PFS as patients with a PET-negative PR, suggesting that patients with a CMR do not require RT following BEACOPP. PET-positive patients who received RT had an inferior prognosis with 2-year PFS of 86% in contrast to 93% in PET-negative patients, suggesting that resistance to chemotherapy is not completely overcome with RT.

These approaches are beginning to be used in clinical practice. However, the 3-year PFS rate in the RATHL trial of 85% for patients with advanced disease was lower than anticipated for patients with interim PET-negative scans, and there is room for improvement in treatment outcomes for patients with advanced disease and interim PET-positive scans.

The role of newer agents including the anti-CD30 antibody–drug conjugate brentuximab vedotin which showed good efficacy in relapsed and refractory disease [90] is currently being explored in the first-line setting in the ‘ECHELON’ trial combined with AVD (https://clinicaltrials.gov/ct2/show/NCT01712490).

In the RATHL trial, the DS in PET-negative patients (DS 1 vs. DS 2 vs. DS 3) did not influence PFS, suggesting that DS 3 is likely to represent CMR with standard treatment. However, it may be prudent to use DS 2 when omission of RT is being considered [49]. It is important therefore to report using the DC and to be aware of the implications of the different scores when patients will be treated using a PET response-adapted approach.

In paediatric HL, the first trial systematically using FDG PET to tailor treatment intensity was the EuroNet-PHL-C1 trial. This trial recruited 2,111 patients with classical HL (all stages) from 18 European countries between 2007 and 2013 [91] (http://clinicaltrials.gov/ct/show/NCT00433459:2012). A Paediatric Hodgkin Network was created to transfer images for central review [92]. Interim PET was performed after two cycles of vincristine, etoposide, prednisone and doxorubicin (OEPA). RT was completely eliminated in patients with an adequate PET response using IHP criteria. The hypothesis was that event-free survival (EFS) would be similar to that of the preceding GPOH-HD-2002 trial using identical chemotherapy in spite of reduced use of RT. RT was omitted in about 50% of patients [77]. The results of the trial are not mature, but publication is anticipated by the end of 2017. In 2015, the EuroNet-PHL-C2 trial started recruitment of paediatric HL patients with 22 countries expected to participate. As in the C1 trial, all patients have interim PET for early response assessment after two cycles of OEPA. In contrast to the C1 trial, for definition of response, the qPET-method is used. A qPET value of ≤1.3 corresponding to a DS of ≤3 is considered an adequate response. It is expected that about 75% of patients will have a DS of ≤3 and will not receive RT.

The COG AHOD0831 trial was the first trial from the Children’s Oncology Group using PET to adapt treatment in HL. It included only patients with very high-risk disease. The IHP criteria were used after one and two cycles. PET-negative patients after two cycles of doxorubicin, bleomycin, vincristine, etoposide, cyclophosphamide and prednisone (ABVE-PC) received standard chemotherapy while slow responders received therapy augmentation with two cycles of ifosfamide/vinorelbine followed by RT to sites of initial bulk. Four-year EFS was 91.9% in PET-negative patients and 87.8% in PET-positive patients after augmented treatment [93].

A response-adapted design has failed to improve patient outcomes in several recent prospective trials in patients with aggressive NHL [94] with the exception of one study presented by the Australian Leukemia and Lymphoma Group [95]. This study enrolled 162 patients with poor prognosis DLBCL. Patients with a PET-positive scan after four cycles of rituximab and cyclophosphamide, doxorubicin, vincristine and prednisone (R-CHOP) were treated with Zevalin and high-dose chemotherapy. Patients with a PET-negative scan continued to six cycles of R-CHOP with two additional rituximab cycles. PFS in PET-positive patients (DS 4 and 5) and PET-negative patients was not statistically significantly different: 2-year PFS was 67% and 74%, respectively (*p* = 0.32), and 2-year OS was 78% and 88%, respectively (*p* = 0.11). In other trials, however, whilst the interim PET result was predictive of patient outcomes in the larger studies presented [96–98], outcomes were not improved using alternative treatment approaches. Unlike HL, in NHL, there are limited options other than the standard treatments of R-CHOP or R-doxorubicin, cyclophosphamide, vindesine, bleomycin and prednisone (R-ACVBP), a more intensive regimen used in younger patients, although trials are now investigating combinations of R-CHOP with immunomodulatory agents such as lenalidomide and idelalisib [99].

## Role in RT planning

PET is becoming increasingly important in planning RT in patients with lymphoma. Treatment of smaller volumes has become possible through better staging of disease with PET and the ability to deliver RT more precisely using modern techniques such as intensity modulated RT (IMRT). A recent review showed that PET modifies the treatment volume in up to 70% of cases [100]. International guidelines now recommend that PET should be used when INRT or involved site RT (ISRT) is employed rather than older IFRT [101, 102].

## Summary

PET is an integral part of monitoring therapy in lymphomas. PET is used to assess remission from disease but more recently has become an important tool to guide response-adapted treatment in adults and children with HL, enabling de-escalation in some patients with reduced toxicity and escalation in others to improve outcomes. PET/CT is used to determine the need for RT and to plan treatment volumes using IMRT enabling a move away from the use of IFRT to ISRT and INRT. The DC have evolved as a standardized method for monitoring disease response, with quantitative approaches in development to further improve the quality and reproducibility of PET response assessment.
